# A comparison of reptilian and avian olfactory receptor gene repertoires: Species-specific expansion of group γ genes in birds

**DOI:** 10.1186/1471-2164-10-446

**Published:** 2009-09-21

**Authors:** Silke S Steiger, Vladimir Y Kuryshev, Marcus C Stensmyr, Bart Kempenaers, Jakob C Mueller

**Affiliations:** 1 Department of Behavioural Ecology & Evolutionary Genetics, Max-Planck Institute for Ornithology, Eberhard-Gwinner-Straße, 82319 Seewiesen, Germany; 2 Institute for Communications Engineering (LNT), Munich University of Technology (TUM), 80290 München, Germany; 3 Department of Evolutionary Neuroethology, Max-Planck Institute for Chemical Ecology, Beutenberg Campus, Hans-Knöll-Straße 8, 07745 Jena, Germany

## Abstract

**Background:**

The detection of odorants is mediated by olfactory receptors (ORs). ORs are G-protein coupled receptors that form a remarkably large protein superfamily in vertebrate genomes. We used data that became available through recent sequencing efforts of reptilian and avian genomes to identify the complete OR gene repertoires in a lizard, the green anole (*Anolis carolinensis*), and in two birds, the chicken (*Gallus gallus*) and the zebra finch (*Taeniopygia guttata*).

**Results:**

We identified 156 green anole OR genes, including 42 pseudogenes. The OR gene repertoire of the two bird species was substantially larger with 479 and 553 OR gene homologs in the chicken and zebra finch, respectively (including 111 and 221 pseudogenes, respectively). We show that the green anole has a higher fraction of intact OR genes (~72%) compared with the chicken (~66%) and the zebra finch (~38%). We identified a larger number and a substantially higher proportion of intact OR gene homologs in the chicken genome than previously reported (214 versus 82 genes and 66% versus 15%, respectively). Phylogenetic analysis showed that lizard and bird OR gene repertoires consist of group α, θ and γ genes. Interestingly, the vast majority of the avian OR genes are confined to a large expansion of a single branch (the so called γ-c clade). An analysis of the selective pressure on the paralogous genes of each γ-c clade revealed that they have been subjected to adaptive evolution. This expansion appears to be bird-specific and not sauropsid-specific, as it is lacking from the lizard genome. The γ-c expansions of the two birds do not intermix, i.e., they are lineage-specific. Almost all (group γ-c) OR genes mapped to the unknown chromosome. The remaining OR genes mapped to six homologous chromosomes plus three to four additional chromosomes in the zebra finch and chicken.

**Conclusion:**

We identified a surprisingly large number of potentially functional avian OR genes. Our data supports recent evidence that avian olfactory ability may be better developed than previously thought. We hypothesize that the radiation of the group γ-c OR genes in each bird lineage parallels the evolution of specific olfactory sensory functions.

## Background

Olfaction plays a pivotal role for many organisms. It is used to detect food, conspecifics, mates, as well as threats. However, the sense of smell is obviously more important to certain animals than others. Birds (with the exception of tube-nosed seabirds, Procellariiformes, and a few members of other groups such as New World vultures or the flightless and nocturnal kiwis) have traditionally been thought to possess an olfactory system inferior to, e.g., mammals, reflecting their overriding reliance on visual and sound cues, exemplified by elaborate and colourful feather plumages (e.g., birds of paradise) and complex song patterns (e.g., nightingales) [for reviews, see [1-3]]. Recent findings have questioned this view, as it turns out that many groups of birds, including songbirds, Passeriformes, apparently have an acute sense of smell and accordingly are likely to also rely on olfaction in their daily life [4-7]. It is important to note that these findings do not imply that all birds have similar olfactory systems and abilities. Instead, one can expect that even closely related species experienced different selection pressures on olfactory abilities. This notion was recently supported by the finding that the olfactory receptor gene repertoire of the nocturnal kakapo, *Strigops habroptilus*, that is known to rely heavily on olfactory cues is larger than that of its related, but diurnal relatives (kea, *Nestor notabilis*, and kaka, *Nestor meridionalis*). This result strongly suggests that e.g. ecological niche adaptations such as daily activity patterns can lead to different olfactory abilities in close relatives [8].

Among vertebrates, the sense of smell is mediated by olfactory receptors (ORs) expressed in sensory neurons within the olfactory epithelium [9,10]. The coding region of OR genes is small (approximately 1000 bp) and intronless [9,11]. Surprisingly, in-silico mining of the first draft of the chicken (*Gallus gallus*) genome (release February 2004) revealed a very large gene family encoding putative ORs [12-14]. Interestingly, the vast majority of these OR genes are confined to a large expansion of a single branch (the so called γ-c clade), in contrast to other vertebrates, where the OR genes are scattered across multiple clades [13,15]. This organization of the chicken OR subgenome was also hinted at in another study on a diversity of nine bird species from seven different orders (Anseriformes, Apterygiformes, Cuculiformes, Galliformes, Passeriformes, Procellariiformes, Psittaciformes), including the chicken [15]. However, the previous study was based on PCR and degenerate primers. Due to limitations of the PCR based method (e.g., primer bias), the previous study may have provided a biased representation of the OR repertoire with respect to the family composition. Thus, it is likely that the results obtained from whole genome searches are more accurate. Genome searches are also needed to show whether the large expansion of OR genes is indeed a common feature of birds and whether it is exclusive to birds within the sauropsid lineage. Finally, a genome-wide approach allows the mapping of the identified OR genes to specific chromosomal locations.

The availability of a second bird genome, that of the zebra finch (*Taeniopygia guttata)*, provides the opportunity for a genome-wide comparative investigation into the structure of the bird OR family. To our knowledge, the OR gene repertoire of this avian model species has not yet been investigated. A genomic analysis of the OR genes from the zebra finch will reveal whether its OR gene repertoire is similar to the previously estimated OR gene repertoires from two other songbirds, that of the blue tit (*Cyanistes caeruleus*) and the canary (*Serinus canaria*) [15]. Furthermore, the recent sequencing of the first reptilian genome (the *Anolis *lizard, *Anolis carolinensis*) allows for a comparison across the sauropsid lineage.

We here report on an exhaustive search for candidate OR genes from the draft genomes of the chicken (release May 2006), zebra finch (release July 2008) and green anole (release February 2007). The use of the recently improved chicken genome assembly improved previous estimates of the OR gene repertoire in the chicken. We identified a larger number and a substantially higher proportion of intact OR gene homologs in the chicken genome than previously reported [13]. We show that the expanded γ-c clade found in chicken is also present in the zebra finch genome. This expansion appears to be bird-specific and not sauropsid-specific, as it is lacking from the lizard genome. We also demonstrate that the γ-c clade has been subjected to adaptive evolution. In addition, and surprisingly, the γ-c expansions of the two bird species do not intermix, i.e., they are lineage-specific. Finally, we show that the green anole has a comparatively small OR gene repertoire compared with other terrestrial vertebrates. Our findings raise the question why birds have evolved a special clade of species-specific OR genes. The function of these genes in relation to the birds' reliance on smell remains unknown.

## Methods

### Detection of OR genes from the genome

The draft genome assemblies of the green anole (*Anolis carolinensis*, released in February 2007; anoCar1), the chicken (*Gallus gallus*, released in May 2006; galGal3) and the zebra finch (*Taeniopygia guttata*, released in July 2008; taeGut1) were downloaded from the UCSC Genome Bioinformatics Site [16]. Note that at the time of our analysis, the green anole genome consisted of 7,233 'scaffold' sequences.

In a first screening local TBLASTN [17] searches with an E-value ≤ 10 were conducted using known amino acid sequences of 3317 intact OR genes from seven species (western clawed frog *Xenopus tropicalis *(410), zebra fish *Danio rerio *(98), puffer fish *Fugu rubripes *(40), chicken (77), mouse *Mus musculus *(1106), rat *Rattus norvegicus *(1198), and human *Homo sapiens *(388)). Sequences were obtained from ref. [13] (western clawed frog, zebra fish, puffer fish, chicken) and [18] (human). Mouse and rat OR sequences were obtained by downloading sequences annotated with the keyword 'olfactory receptor' from GenBank [19].

After collecting hit sequences of putative OR genes, non-overlapping blast-hits showing the lowest E-values were extracted. A local version of RepeatMasker [20] that contained either (i) a specific library of known chicken full-length OR nucleotide sequences [13] or (ii) a (non-OR) G-protein coupled receptor (GPCR) sequence library consisting of 327 chicken sequences [14] was used to distinguish OR genes from non-OR GPCRs. Sequences that were more similar to non-OR GPCRs than to OR genes or shorter than 150 nucleotides were removed from further analyses. Remaining hits were subsequently classified into intact genes, partial genes or pseudogenes following reference [18] with minor modifications. In brief, translated sequences were regarded as "intact" if they were ≥ 250 amino acids long, included the start codon methionine and comprised all seven transmembrane (TM) domains without any interrupting stop codons and/or frameshifts. TM domains were identified using the TMHMM server [21,22]. Therefore, intact OR genes are potentially functional and likely to be expressed on olfactory receptor neurons. Sequences were regarded as "pseudogenes" if a stop codon and/or frameshift and/or less than seven TM domains were detected by visual inspection of the alignment. Sequences were regarded as "partial" if they were shorter than 750 nucleotides and contained at least one sequence gap within the sequence or its 20 nucleotide-long flanking region. Open reading frames (ORFs) were detected and extracted using the program *getorf *[23] of the EMBOSS package [24]. Sequences that were identified and classified as "intact" were added to the RepeatMasker library. Subsequently, a second round of analysis was performed to ensure that the search was exhaustive. All OR genes were mapped to the corresponding genomic sequences.

We used the program "skipredundant" of the EMBOSS package [24,25] to detect redundant hits in our sets. We only included OR genes whose sequences and flanking regions (up to 500 nucleotides) were not identical.

### Phylogenetic analyses

The amino acid sequences of intact OR genes were used for the construction of a multi-species phylogenetic tree (see below). MAFFT [26,27] was used with default settings to construct multiple amino acid sequence alignments of intact OR genes. GENEDOC [28,29] was used for visual inspection and manual correction of alignments (e.g., trimming of N- and C-highly variable ends). Phylogenetic trees were constructed from Poisson corrected distances using the Neighbor-Joining (NJ) method implemented in the MEGA 4 software [30,31]. The reliability of the phylogenetic trees was evaluated with 1000 bootstrap replicates. HyperTree [32] was used to edit the phylogenetic trees.

### Detection of conserved motifs

To detect conserved motifs in predicted OR protein sequences, sequence logos were generated from an alignment of intact green anole, chicken and zebra finch OR sequences using the program WebLogo [33,34]. The TMHMM server [21] was used to identify intracellular (IC) and extracellular (EC) domains.

### Positive selection analyses

Selection at the protein level can often be detected using the ratio of the rate of nonsynonymous to the rate of synonymous substitutions (ω = *d*_*N*_/*d*_*S*_) [35]. Positive selection (i.e., selection favoring changes in the protein sequence), neutral evolution and purifying selection (selection against deleterious alleles and thus, against changes in the protein sequence), is indicated by ω>1, ω = 1 and ω<1, respectively [35]. The single likelihood ancestor counting (SLAC) method with default settings, implemented in the Datamonkey web-interface [36] was used to estimate the global ω value and to test for signatures of positive selection on individual codons of each group γ-c clade (see below). The intact OR sequences were codon-aligned using MAFFT, the alignment was manually edited and alignment gaps present in >85% of the sequences were removed. A significance level of 0.05 was used. Because the SLAC analysis is currently restricted to 150 sequences [36], we randomly selected 150 out of 165 chicken group γ-c OR genes for analysis. Note that although these methods are generally capable of efficiently identifying positively selected sites, they may also miss some positively selected sites, in particular if positive selection is weak.

## Results

### Composition of the green anole, chicken and zebra finch OR gene repertoires

OR genes from the draft genome assemblies of the green anole, zebra finch and chicken were identified by a comprehensive data mining approach. The numbers of intact, partial and pseudogenes that were identified in the three species are shown in Table 1. The amino acid sequences of the intact OR genes and the nucleotide sequences of pseudogenes identified in this study are available in the supplementary material (see Additional file 1 &2).

**Table 1:** Intact, non-functional (pseudogenes) and partial OR genes in one reptile and two bird species

	**Intact^a^**					**Pseudogenes^b^**					**Partial^c^**						
			
**Species**	**α**	**γ**	**γ-c**	**θ**	**Total**	**α**	**γ**	**γ-c**	**θ**	**Total**	**α**	**γ**	**γ-c**	**θ**	**Total**	**Total^d^**	**% intact** **OR genes** **(range)^e^**
Green anole	1	108	0	1	110	0	42	0	0	42	0	4	0	0	4	156	72 (71;73)
Chicken	9	39	165	1	214	6	26	79	0	111	2	11	141	0	154	479	66 (45;77)
Zebra finch	2	3	128	1	134	3	4	214	0	221	0	3	195	0	198	553	38 (24;60)

a A sequence is defined as "intact" if it possesses a full-length OR protein coding sequence that is at least 250 amino-acids long and has seven TM domains.

b A sequence is defined as "pseudogene" if it possesses at least 1 premature stop codon and/or frameshift and/or has less than seven TM domains.

c A sequence is classified as a partial gene if it is shorter than 250 amino-acids and contains at least one sequence gap on the flanking genomic region.

d Numbers indicate the sum of intact genes, pseudogenes and partial genes.

e The percentage of intact OR genes is calculated as the ratio of 100*intact genes/(intact genes + pseudogenes). Numbers in brackets indicate the proportion of intact OR genes assuming that all partial genes will turn out to be pseudogenes (first number) or intact OR genes (second number).

The proportion of intact OR genes varied between 38% (zebra finch) and 72% (green anole) (Table 1). The entire OR gene repertoires were of similar size in the chicken and the zebra finch (~500), but the chicken had a larger number (and thus proportion) of intact OR genes (Table 1). Approximately 30% of the avian OR repertoires were partial sequences. Improved versions of the genomes will clarify whether these sequences are pseudogenes or intact OR genes.

The avian OR gene repertoires were approximately three times larger than that of the green anole (Table 1). A comparison with Niimura & Nei's [13] and Lagerstrom's [14] datasets revealed that we identified 160 novel chicken OR genes (30 intact, 86 partial and 44 pseudogenes) that were not reported previously.

### Phylogenetic relationships of green anole, zebra finch and chicken OR genes

**Figure 1:**
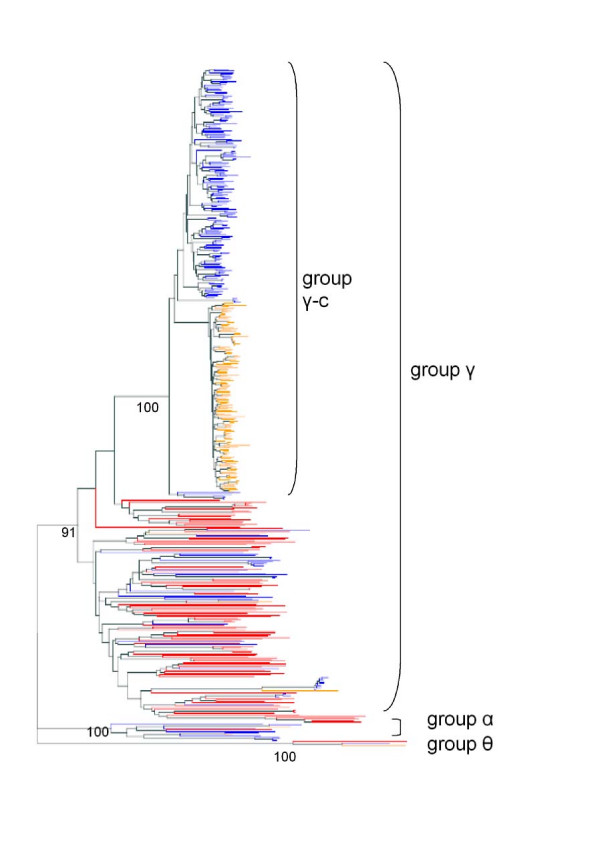
**Phylogenetic tree of sauropsid OR genes**. Unrooted NJ tree derived from aligned green anole (red lines, 112 sequences), chicken (blue lines, 214 sequences) and zebra finch (orange lines, 134 sequences) predicted full-length OR protein sequences. Bootstrap values were obtained by 1000 replicates, and the values are shown for the major clades.

All putative ORs appeared to be bona fide ORs when evaluated. Following Niimura & Nei's classification [13], lizard and bird OR gene repertoires consist of group α, θ and γ genes. The large majority of OR genes in the green anole, chicken and zebra finch can be classified as group γ OR genes (equivalent to the "class II" genes [37]) (Figure 1). Several clades contained both bird and green anole OR sequences, indicating that these clades diverged before the divergence of the three species. Interestingly, there was an enormous expansion in the number of genes in the γ-groups of both bird species, termed the γ-c clade (Figure 1). This clade was supported by a high bootstrap value (100%). This expansion seems to be specific to birds, as it could not be detected in the green anole (Figure 1) and in other vertebrates (data not shown)[15,37]. Approximately 77% and 96% of the intact genes in chicken and zebra finch were group γ-c genes. Notably, within the group γ-c ORs, sequences from the species did not intermingle with each other. Members of the γ -c clade were highly similar in sequence (on average 88 and 92% sequence identity on the nucleic acid level in the chicken and zebra finch, respectively; see below). An analysis of the selective pressures on the paralogous genes of each group γ-c clade revealed that the global ω values were similarly low for both bird species (0.44 and 0.45 in the chicken and zebra finch, respectively), indicating that most avian group γ-c clade codons are under purifying selection. The site-by-site analysis showed that there is evidence for positive selection at some sites: the SLAC software detected 16 (zebra finch) and 18 (chicken) positively selected codons, respectively (see Additional File 3). In both species, seven positively selected codons were located in TM regions (see Additional file 3).

**Figure 2:**
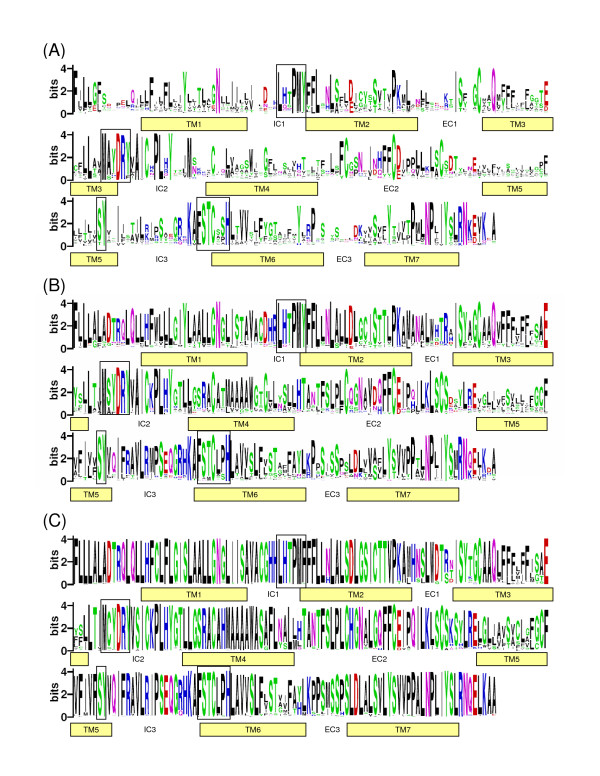
**Amino acid sequence conservation in the reptilian and avian OR gene repertoires**. Sequence logos of intact (A) green anole, (B) chicken and (C) zebra finch OR sequences. The logos were generated from an alignment of 110, 214, and 134 predicted full-length OR sequences from the green anole, chicken and zebra finch using the program WebLogo. Heights of amino acid letters represent the relative frequency at a given position. The overall height at a given position indicates the level of sequence conservation. Bars below amino acids indicate transmembrane regions (TM). Note that the exact number and precise placement of the TMs has not been experimentally verified and should thus be treated with caution. The position of intracellular (IC) and extracellular (EC) domains are also indicated. Black boxes indicate four conserved motifs that are characteristic for vertebrate ORs.

An overview of sequence identities between green anole, chicken and zebra finch intact OR genes is provided in Additional file 4. Sequence logos of predicted green anole, chicken and zebra finch protein OR sequences illustrate the sequence conservation of ORs (Figure 2). Notably, avian ORs were generally more conserved than those from the green anole. This is indicated by fewer and larger letters at individual positions in the logo. The main reason for this observation is that the members of the expanded γ-c clade in birds are highly identical in sequence (the majority of OR genes belong to the γ-c clade in birds, see above). Four conserved motifs that are characteristic for ORs and have been described in other vertebrate species [for review, see ref [38]] were also found with slight modifications in the three species investigated in this study (Figure 2). Notably, one feature was quite distinct between the three species: the consensus motif MAYDRY was found in the green anole, whereas the motif MSYDRYand MCYDRY is characteristic for chicken and the zebra finch ORs, respectively.

### Genomic locations of green anole, chicken and zebra finch OR genes

The genomic locations of chicken and zebra finch OR genes are shown in Table 2. OR genes were distributed on 10 and 9 chromosomes in the chicken and zebra finch, respectively. In the chicken and zebra finch, OR genes were also mapped to one linkage group. ORs were distributed on macro-chromosomes (chicken: chromosome 1, 5; zebra finch: 1, 2, 5), intermediate chromosomes (chicken: chromosome 9, 10; zebra finch: 9, 10) and micro-chromosomes (chicken: chromosomes 11, 17, 18, 25, 27; zebra finch: 17, 27) (the classification of chromosomes follows ref [12]). Group α and θ genes occur on homologous chromosomes in both avian genomes. In the chicken only, one pseudogene was mapped to a sex chromosome. However, the large majority of chicken and zebra finch OR genes (81 and 91%, respectively) were assigned to the unknown chromosome (chrUn_random and Un in the chicken and zebra finch, respectively), which refers to sequence contigs that are not yet allocated to named chromosomes. In particular, the chromosomal location of almost all intact group γ-c OR genes is still unknown (156 out of 165 in the chicken and 116 out of 128 in the zebra finch, respectively; Table 2). Group γ-c OR genes that were assigned to the unknown chromosome could be localised on 129 and 362 distinct supercontigs (i.e., an ordered group of contigs that still include gaps) in the chicken and zebra finch, respectively (out of 17001 and 35035 possible supercontigs, Table 3). We could not determine on how many chromosomes green anole OR genes were distributed because the genome information is currently only organized in scaffolds. Yet, anole OR genes were distributed in only 20 scaffolds of 7233 possible, strongly suggesting that green anole OR genes occur in clusters in the genome.

**Table 2:** Chromosomal location of OR genes in chicken and zebra finch

**Species**	**Chromosomal**	**Intact**					**Pseudogenes**					**Partial**				
	**Location**	**α**	**γ**	**γ-c**	**θ**	**Total**	**α**	**γ**	**γ-c**	**θ**	**Total**	**α**	**γ**	**γ-c**	**θ**	**Total**
**Chicken**	1	9	2	0	0	11	6	1	0	0	7	0	0	0	0	0
	5	0	15	0	0	15	0	8	0	0	8	0	3	0	0	3
	9	0	0	0	1	1	0	0	0	0	0	0	0	0	0	0
	10	0	7	0	0	7	0	9	0	0	9	0	1	0	0	1
	11	0	0	0	0	0	0	0	1	0	1	0	0	0	0	0
	17	0	1	0	0	1	0	0	1	0	1	0	0	0	0	0
	18	0	0	0	0	0	0	0	1	0	1	0	0	0	0	0
	25	0	0	0	0	0	0	0	1	0	1	0	0	0	0	0
	27	0	2	0	0	2	0	3	0	0	3	0	0	0	0	0
	Z	0	0	0	0	0	0	1	0	0	1	0	0	0	0	0
	chrUn_random	0	12	156	0	168	0	4	70	0	74	2	7	139	0	148
	chrE22C19W28_E50C23_random^a^	0	0	9	0	9	0	0	5	0	5	0	0	2	0	2
																
**Zebra**	1	0	1	0	0	1	0	0	0	0	0	0	0	0	0	0
**finch**	1A_random^b^	0	0	0	0	0	0	0	0	0	0	0	2	0	0	2
	1B_random^b^	2	0	0	0	2	3	0	0	0	3	0	0	0	0	0
	2	0	0	2	0	2	0	0	3	0	3	0	0	2	0	2
	5	0	1	0	0	1	0	2	0	0	2	0	1	0	0	1
	9	0	0	0	1	1	0	0	0	0	0	0	0	0	0	0
	10_random^b^	0	0	1	0	1	0	0	4	0	4	0	0	0	0	0
	17_random^b^	0	0	2	0	2	0	0	0	0	0	0	0	0	0	0
	27	0	0	1	0	1	0	1	2	0	3	0	0	1	0	1
	Un	0	1	116	0	117	0	1	198	0	199	0	0	188	0	188
	LGE22_random^a^	0	0	6	0	6	0	0	7	0	7	0	0	4	0	4

a Linkage group (a collection of supercontigs on the same chromosome)

b genomic sequences that are assigned to, but not located on a chromosome.

**Table 3:** Distribution of group γ-c OR genes that were assigned to the unknown chromosome

**Species**	**No. supercontigs**	**No. supercontigs encoding group **γ **-c OR genes**	**No. group **γ **-c OR genes**	**Ratio (No. group **γ **-c OR genes/supercontig)**
Chicken	17001	129	365	2.8
Zebra finch	35035	362	502	1.4

## Discussion

### Comparison of the reptilian and the avian OR gene repertoires

We showed that the OR gene repertoires are substantially larger in the two bird species (chicken, zebra finch) than in the green anole. Similarly, the absolute number of intact OR genes varied greatly, with twice as many intact OR genes in the chicken than in the green anole. The number of intact OR genes was only slightly larger in the zebra finch than in the green anole. However, we expect the real number of intact OR genes in the zebra finch to be larger, because many partial genes may turn into intact genes in the next assembly of the zebra finch genome.

It is reasonable to assume that gene duplications occurred in the bird lineages after the reptile-bird divergence, possibly as an adaptation to new ecological niches and new odorous environments [39]. As an intermediate evolutionary step, copy number variations within populations may have contributed to the intensive paralog birth in the bird lineage [40,41]. The "successful" paralogs may have been fixed and maintained, whereas "unsuccessful" paralogs became pseudogenes in the population [42]. Interestingly, copy number variations are specifically enriched among evolutionary "young" OR genes in humans (i.e., human ORs that have a closely related paralog in the human genome) [40]. It is possible that the same mechanism applies to the recently expanded avian group γ-c genes.

The group γ-c OR genes rapidly expanded in the chicken and zebra finch lineage, but is absent in the anolis lineage and in other vertebrates. Within the γ-c OR clade, sequences from the same species were very similar and therefore cluster together in phylogenetic trees. Therefore, this result supports a previous study that was based on PCR and degenerate primers, rather than on genomic data [15]. Two different scenarios that are not mutually exclusive may explain this clustering pattern. First, the species-specific γ-c OR clades may have arisen from independent expansion events. Second, ancient γ-c OR clade genes became homogenized by concerted evolution within species [15,43]. Because even a single point mutation in the binding site of an OR can alter the ligand specificity thereby increasing or decreasing the affinity of the OR for certain odorant molecules [44], a large number of paralogs could be advantageous, for example in evolutionary arms races between predator and prey.

It has been suggested that positive selection contributes to a diverse repertoire of OR genes in fish [[45-47], but see, [48]] and mammals [[49-53], but see, [54,55]]. We showed that a large number of positively selected sites are present in group γ-c OR genes and thus, it is likely that similarly, positive selection also contributes to a diverse repertoire of OR genes in birds. In both bird species, seven positively selected codons were located in TM domains. Furthermore two (chicken) and three (zebra finch) of these seven codons were located in TM5, a domain which forms much of the putative ligand-binding pocket of OR receptors [56]. Therefore, these group γ-c OR codons that show signatures of positive selection are likely to be functionally relevant. Average ω values usually range from 0.05 to 0.25 in other vertebrates. Hence, the estimates presented here in both bird species (~0.4) were high. This can be explained by the occurrence of a large number of sites under positive selection. A similar pattern has been observed in trace amine-associated receptor (TAAR) genes, a second class of chemosensory receptors that are expressed in the olfactory epithelium [57-59]. One has to keep in mind that the results of the positive selection test should be treated with caution because estimates of positive selection among members of multi-gene families may be flawed if there are homogenizing effects caused by gene conversion within the family [60]. As mentioned above, potential concerted evolution among the γ-c OR genes within species could be interpreted in such that gene conversion is common. Ideally, functional validation of the ORs to test whether the results are biologically significant should be conducted [61].

It is tempting to speculate that the species-specific clustering of the γ-c clade reflects species-specific chemosensory capacities. However, to our knowledge, experimental evidence supporting this hypothesis is currently lacking. Interestingly, a similar rapid expansion of OR genes has been observed in the western-clawed frog (*Xenopus tropicalis*) lineage (this clade was termed γ-a and γ-b) [13]. It may be worth to investigate whether this expansion occurred in other amphibians as well, or whether it is restricted to *Xenopus tropicalis*.

### Chicken olfactory receptor genes

The total number of chicken OR genes was similar to those reported in previous studies [[13,39] but see reference [12]] (see Additional file 5). However, our estimate of the proportion of intact OR genes in the chicken was substantially higher than previously reported [13](but see ref [12,39]). The most likely reason for this discrepancy is that we used a more recent and improved version of the chicken genome assembly (Version 2.1; released in May 2006). As predicted by Niimura and Nei [13], the quality of the second draft of the chicken genome increased and the number of short contigs that are few kilobases long decreased. Therefore, it is not surprising that we could identify a larger number of intact OR genes. Due to the existence of 154 partial genes that may become intact in the next assembly of the chicken genome, we may still have underestimated the number of intact OR genes.

### Comparison of the chicken and zebra finch OR gene repertoires

Previously, we used PCR with degenerate primers and a non-parametric estimation technique to assess OR gene repertoire sizes in nine different bird species from seven different orders [15]. Based on our results, we hypothesized that the chicken would have a larger OR gene repertoire but a similar fraction of functional OR genes than the zebra finch. Contrary to our hypothesis, the OR gene repertoires - identified by searching the genome databases - were of similar size (~500) and the fraction of intact OR genes were higher in the chicken than in the zebra finch (66 versus 38%). However, one has to keep in mind that the proportion of intact OR genes in the zebra finch is probably underestimated, as argued above. Hence, we expect that forthcoming and improved versions of the genomic sequences will lead to an increase in the estimated proportion of intact OR genes (as observed in the chicken).

Comparative genomic studies suggest that the olfactory acuity of vertebrates correlates positively with the proportion of intact OR genes encoded in their genomes [62]. For example, Gilad et al. [62] showed that New World monkeys and prosimians - animals that highly rely on their sense of smell - have a higher proportion of intact OR genes than vision-oriented Old world monkeys and apes that have evolved a trichromatic colour-vision system. In addition, amongst primates, the proportion of intact OR genes is significantly reduced in humans (~50%) when compared with other apes (~70%) [[62,63], but see [64]]. Accordingly, one may expect the zebra finch to have reduced olfactory capabilities compared to the chicken. This seems reasonable because the relative size of the olfactory bulb compared to the cerebral hemisphere, often used as a morphological indicator of olfactory ability, is considerably smaller in the zebra finch than in the chicken (4 versus 15%, respectively)[65]. Therefore, songbirds were long thought to not have a well-developed sense of smell. Nevertheless, evidence is accumulating that songbirds use their sense of smell in a variety of contexts [4,6,66]. Therefore, we agree with Nei et al. [39] and doubt that the proportion of intact OR genes is a good indicator of olfactory abilities

### Chromosomal location of OR genes

Previous studies showed that OR genes are distributed on different chromosomes and generally form genomic clusters in vertebrates [39,67,68]. Although we could only assign few OR genes to chromosomes, there is evidence that OR genes occur on at least 9 and 10 chromosomes in the zebra finch and the chicken (out of 40 and 39, respectively). The distribution of OR genes on the chromosomes was generally well conserved between the chicken and the zebra finch. This may not be surprising because the conservation of the avian karyotype is relatively high [69]. The distribution of the γ-c OR gene clade could not been determined. We doubt that their multiple placement on "unknown" chromosomes is simply an assembly artefact because Southern Blots using group γ-c OR genes as probes reveal a large number of bands in several bird species [8], and chicken [8], unpublished data]. It seems reasonable to assume that γ-c OR genes are distributed on only few chromosomes for two reasons. First, OR genes that cluster together in a phylogenetic tree usually cluster in chromosomal locations (see above). Second, the number of supercontigs encoding group γ-c OR genes are considerably smaller than the total number of OR genes. However, it should be noted that currently there seems to be more evidence for genomic clustering of OR genes in the chicken than in the zebra finch. In addition, it is likely that γ-c OR genes are distributed on micro-chromosomes rather than on macro-chromosomes for two reasons. First, whereas the macro-chromosomes have been sequenced with high coverage, the micro-chromosomes are still poorly covered [70]. Second, estimates of nucleotide substitution and recombination rates are higher on micro-chromosomes than on macro-chromosomes [71] which might explain the rapid expansion of group γ-c paralogs. Improved versions of the genomes investigated may yield additional insights about the genomic distribution of OR genes, in both reptiles and birds.

## Conclusion

The large number of potentially functional avian OR genes supports the notion that avian olfactory ability may be better developed than previously thought, and perhaps even better developed than in reptiles. We hypothesize that the radiation of the group γ-c OR genes in each bird lineage parallels the evolution of specific olfactory sensory function, but this remains to be shown.

## List of abbreviations used

chrUn: chromosome unknown; EC: extracellular domain; GPCR: G-protein coupled receptor; IC: intracellular domain; OR: olfactory receptor; TM: transmembrane domain.

## Authors' contributions

SSS initiated the study and wrote the manuscript. VYK performed the bioinformatic analyses. VYK and MCS contributed to the writing of the paper. BK and JCM contributed to the critical revision of the manuscript. JCM conceived the study and guided the project. All authors participated in the interpretation and discussion of results. All authors read and approved the final manuscript.

## Supplementary Material

Additional file 1 **Amino acid sequences of OR genes**. Amino acid sequences of intact OR genes identified in three vertebrate genomes (green anole, chicken, zebra finch). Click here for file

Additional file 2 **Nucleotide sequences of pseudogenes**. Nucleotide sequences of pseudogenes identified in three vertebrate genomes (green anole, chicken, zebra finch). Click here for file

Additional file 3 **Positively selected codons**. Sequence logo of (A) chicken and (B) zebra finch group γ-c ORs indicating positions of positively selected codons. The X axis indicates the amino acid position while the symbol height (Y axis) indicates the relative frequency of each amino acid at that position. Predicted transmembrane domains (TM), intracellular (IC) and extracellular (EC) domains are indicated. Asterisks above the amino acids indicate sites that were predicted to be positively selected. Click here for file

Additional file 4 **Sequence identities**. Sequence identities (in %) between green anole, chicken and zebra finch OR genes on the nucleic acid level. Numbers in brackets indicate the minimum and maximum pairwise identities. Abbreviation: N.A. = not applicable. Click here for file

Additional file 5 **OR genes identified from the chicken genome**. Comparison of the numbers of intact OR genes, pseudogenes and partial OR genes estimated from the chicken (*Gallus gallus*) genome. Abbreviation: N.D. = not determined. Click here for file
