# Supplementary figures and images for: A comparison of reptilian and avian olfactory receptor gene repertoires: Species-specific expansion of group γ genes in birds
Source: BMC Genomics. 2009 Sep 21;10:446. doi: 10.1186/1471-2164-10-446 (PMC2758906; doi:10.1186/1471-2164-10-446)

(A)

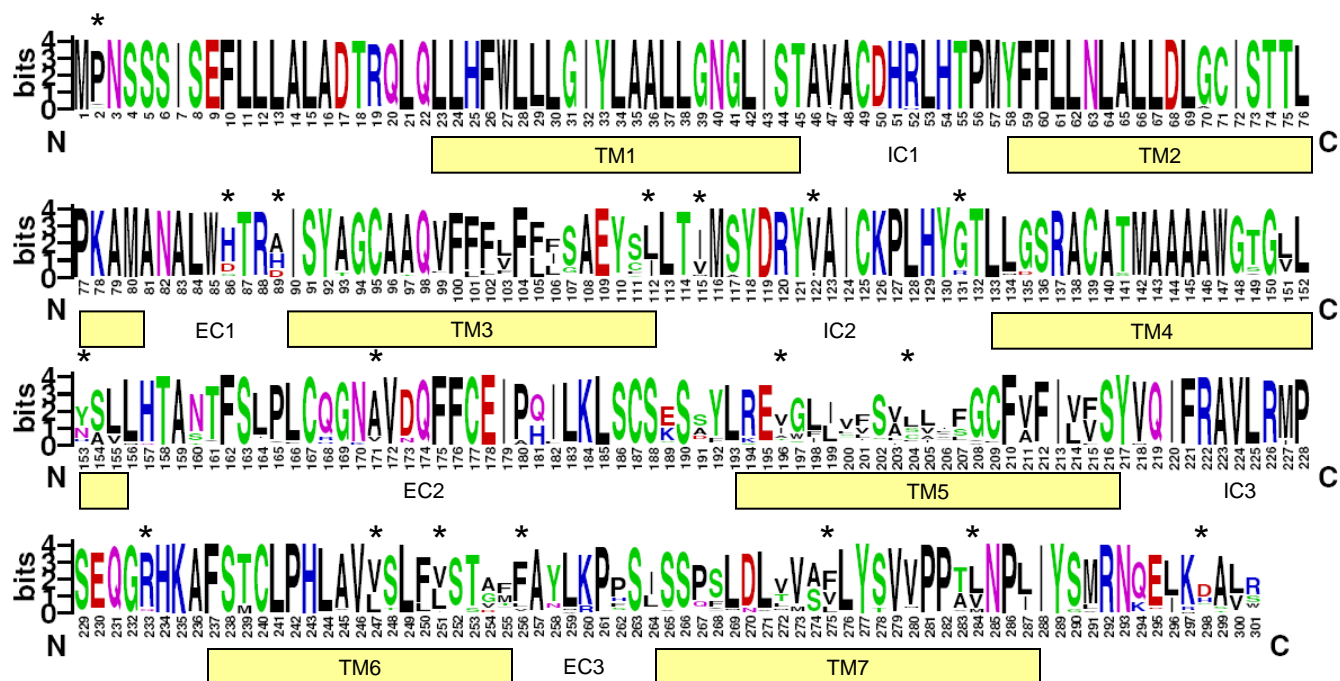

(B)

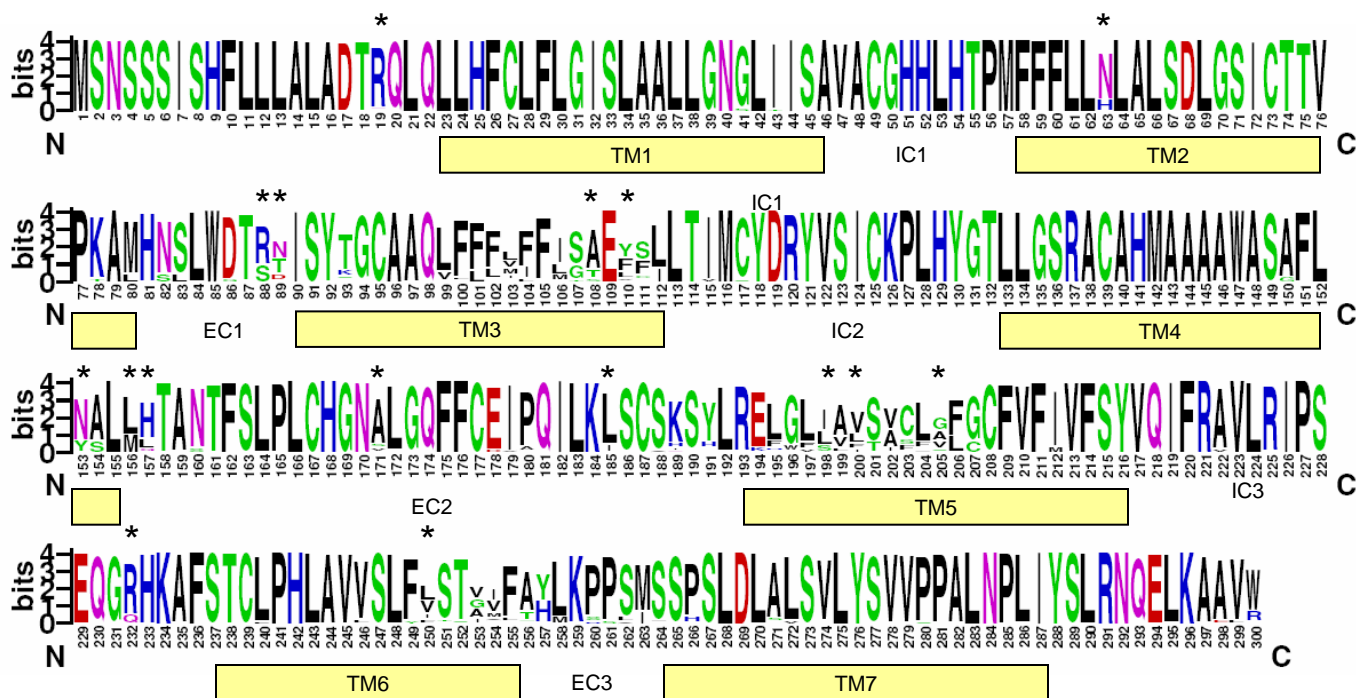

Supplement: Additional file 3 — Positively selected codons. Sequence logo of (A) chicken and (B) zebra finch group γ-c ORs indicating positions of positively selected codons. The X axis indicates the amino acid position while the symbol height (Y axis) indicates the relative frequency of each amino acid at that position. Predicted transmembrane domains (TM), intracellular (IC) and extracellular (EC) domains are indicated. Asterisks above the amino acids indicate sites that were predicted to be positively selected. [file 1471-2164-10-446-S3.PDF]
