# Supplementary material for: A comparison of reptilian and avian olfactory receptor gene repertoires: Species-specific expansion of group γ genes in birds
Source: BMC Genomics. 2009 Sep 21;10:446. doi: 10.1186/1471-2164-10-446 (PMC2758906; doi:10.1186/1471-2164-10-446)
Supplement: Additional file 4 — Sequence identities. Sequence identities (in %) between green anole, chicken and zebra finch OR genes on the nucleic acid level. Numbers in brackets indicate the minimum and maximum pairwise identities. Abbreviation: N.A. = not applicable. [file 1471-2164-10-446-S4.DOC]

**Additional File 4 – Average sequence identities (in %) between intact green anole, chicken and zebra finch OR genes on the nucleic acid level (range in brackets).**

|  | **Green anole** | | | | **Chicken** | | | | **Zebra finch** | | | |
| --- | --- | --- | --- | --- | --- | --- | --- | --- | --- | --- | --- | --- |
|  | **α** | **γ** | **γ -c** | **θ** | **α** | **γ** | **γ -c** | **θ** | **α** | **γ** | **γ -c** | **θ** |
| Green anole | N.A. | 52 (42-100)^a^ | N.A. | N.A. |  | | | |  | | | |
| Chicken | 57 (53-67) | 51 (44-72) | N.A. | 64 | 61 (54-97) | 56 (46-99) | 88 (79-99) | N.A. |  | | | |
| Zebra finch | 61 (61-62) | 49 (43-66) | N.A. | 65 | 63 (57-88) | 53 (47-79) | 83 (77-86) | 79 | 63 | 50 (47-55) | 92 (88-99) | N.A. |

^a^ in one case, a sequence was 100% identical to another sequence but the flanking regions differed. Thus, we counted each of these sequences as distinct OR gene.
