# Supplementary material for: A comparison of reptilian and avian olfactory receptor gene repertoires: Species-specific expansion of group γ genes in birds
Source: BMC Genomics. 2009 Sep 21;10:446. doi: 10.1186/1471-2164-10-446 (PMC2758906; doi:10.1186/1471-2164-10-446)
Supplement: Additional file 5 — OR genes identified from the chicken genome. Comparison of the numbers of intact OR genes, pseudogenes and partial OR genes estimated from the chicken (Gallus gallus) genome. Abbreviation: N.D. = not determined. [file 1471-2164-10-446-S5.DOC]

**Additional File 5 – OR genes identified from the chicken genome**

|  | **International Chicken Genome Sequencing Consortium, 2004** | **Niimura & Nei, 2005** | **Lagerstrom et al., 2006** | **Niimura et al., 2008** | **This study** |
| --- | --- | --- | --- | --- | --- |
| Chicken genome  assembly | Version 1.1  (February 2004) | Version 1.1  (February 2004) | Version 1.1  (February 2004) | Version 2.1  (May 2006) | Version 2.1  (May 2006) |
| Intact genes | 202^a^ | 82 | 148 | 300^d^ | 214 |
| Partial- / pseudogenes | N.D.^e^ / 16^a^ | 476^c^ | 81 / N.D.^e^ | 133 | 154 / 111 |
| Total | 283 ^b^ | 558 | N.D.^e^ | 433 | 479 |
| % Intact | N.D.^e^ | 15 | N.D.^e^ | 69 | 66 |

^a^ group γ-c members

^b^ unclear whether pseudogenes are included

^c^ both partial and pseudogenes

^d^ including partial genes

^e^ N.D. = not determined
